# Immediate functional loading of single implants: a multicenter study with 4 years of follow-up

**DOI:** 10.15171/joddd.2018.005

**Published:** 2018-03-14

**Authors:** Filiep Raes, Tammaro Eccellente, Carolina Lenzi, Michele Ortolani, Giuseppe Luongo, Carlo Mangano, Francesco Mangano

**Affiliations:** ^1^Professor, Department of Periodontology and Oral Implantology, University of Ghent, Belgium; ^2^Private Practice, Grumo Nevano, Naples, Italy; ^3^Private Practice, Bologna, Italy; ^4^Private Practice, Naples, Italy; ^5^Professor, Department of Oral and Maxillofacial Surgery, Dental School, University of Naples, Italy; ^6^Professor, Department of Dental Sciences, University Vita Salute San Raffaele, Milan, Italy; ^7^Lecturer, Department of Surgical and Morphological Science, Dental School, University of Varese, Italy

**Keywords:** Dental implants, endosseous, Immediate loading, post-operative complications, survival analysis

## Abstract

***Background.*** In the current scientific literature there are only few studies on the immediate functional loading of single implants. The aim of the present present study was to evaluate the 4-year survival rate, complication rate and peri-implant marginal bone loss (PIMBL) of immediately loaded single implants inserted in healed ridges and fresh post-extraction sites.

***Methods.*** Six centers were involved in this prospective study. The surgical and prosthetic protocol was defined in detail, before the start of recruiting patients. Recruitment of patients and performance of surgeries took place between February 2012 and February 2013. Criteria for inclusion were single-tooth gaps in healed ridges and fresh post-extraction sockets. All the fixtures (Anyridge®, Megagen Corporation, Gyeongbuk, South Korea) were functionally loaded immediately after insertion and followed for a period of 4 years. Outcome measures were implant survival, complications and PIMBL.

***Results.*** Forty-six patients (18‒73 years of age) were selected. In total, 57 fixtures were placed (10 in fresh post-extraction sockets). After 4 years of functional loading, only one fixture was lost; therefore, high survival rates (97.6% patient-based; 98.1% implant-based) were reported. In addition, a limited incidence of biologic (4.8% patient-based; 3.8% implant-based) and prosthetic (9.7% patient-based; 7.6% implant-based) complications was reported. The overall 4-year PIMBL amounted to 0.38±0.21 mm (healed ridges: 0.4±0.21 mm; fresh post-extraction sockets: 0.33±0.20 mm).

***Conclusion.*** Loading single implants immediately seems to be a highly successful treatment modality. However, long-term data are needed to confirm these positive outcomes.

## Introduction

Dental implants today represent a reliable and successful solution for the rehabilitation of single tooth gaps, as unequivocally demonstrated by literature in the short,^1^ medium^2^ and long term.^3^

The success of prosthetic implant therapy depends essentially on the biological, functional and aesthetic integration of implants and implant-supported prosthetic restorations in the oral cavity.^4,5^ Today, in order to meet the needs and demands of patients, it is no longer sufficient that a properly osseointegrated implant remain functional for many years; it is also important that the aesthetic integration of the implant-supported prosthetic restoration be fully satisfactory.^6,7^

However, patients are increasingly more demanding and the demand for a satisfactory aesthetic result with a fully biomimetic rehabilitation in the oral cavity is today accompanied by the desire to reduce treatment times and replace a missing or irreparably compromised natural tooth with a definitive implant-supported restoration as early as possible.^6,8^

In this context, solutions such as immediate implant placement in post-extraction sockets,^6,8,9^ where there is a compromised dental element that requires replacement, and immediate loading^10-12^ are increasingly successful, required by patients and provided by dentists.

Immediate placement of an implant involves inserting a fixture into a post-extractive alveolus, immediately after extracting a tooth which is no longer recoverable because it is compromised.^6,8,9,11^ Patients like this solution because it reduces surgical sessions and psychological stress; it is a classic ‘two-for-one’ deal, ensuring minimal invasiveness and a significant reduction in implant treatment times.^6,8,9,11^

A further reduction in treatment time is then provided by immediate loading, i.e., by the possibility of immediate functionality of the implant by positioning, within 48‒72 hours after fixture placement, a provisional restoration on it.^10-13^ Immediate loading allows one to significantly reduce the time of prosthetic treatment, since the functionality of the implant is immediate and the healing times originally indicated in classical implantology, which ranged from 4 to 6 months, are thereby eliminated.^10-13^ This also eliminates the need for troublesome temporary prostheses, whether removable or cemented onto adjacent teeth, with less stress and discomfort and greater patient satisfaction.^10-13^

Although the advantages of immediate loading are well known in the literature^10-13^ and this procedure is now regarded as reliable and successful in various clinical contexts,^10-16^ it is mandatory to remember how, in immediate loading, it is crucial to achieve primary stability (i.e. mechanical stabilization of the fixture at the time of the positioning) as well as a secondary stability (i.e. a biological stabilization of the fixture in the first healing period), both of which being optimal.^17,18^

If, in fact, the surgeon is unable to obtain adequate primary stabilization of the implant, immediate loading and transmission of force from occlusion and tissues (tongue, cheeks) through prosthetic restoration can in fact lead to mobility and therefore to the loss of the fixture, as classically described in the literature.^5,10-18^ Beyond a certain threshold, in fact, the presence of micromotions at the bone‒implant interface may interfere with osseointegration and determine the failure of implant therapy.^16-20^ In order to obtain a proper integration of the implant under load in the first healing period (the first 2–3 months after insertion), it is of crucial importance that new bone be rapidly deposited on the implant surface.^21-23^ This allows for a secondary (biological) stabilization of the implant, one that will counteract and balance the reduction of primary stabilization that physiologically occurs when bone remodeling begins.^21-23^ If this is not the case, there is a risk that the implant will fail.^23,24^

Since the number of forces transmitted from the prosthesis to the bone-implant interface in the first healing period is an important element and can even determine the failure of an implant procedure, it is essential to distinguish between immediate functional^10-14,25^ and non-functional^13,16,25,26^ loading.

In immediate functional loading, the implant restoration features all static and dynamic occlusal contacts, and therefore transmits the chewing forces to the fixture entirely, as well as the forces derived from muscles (e.g. the tongue and cheeks).^10,13,25^

In immediate non-functional loading, instead, prosthetic restoration can be abundantly discharged by chewing, effectively eliminating static and dynamic occlusal contacts, in order to provide a safer healing period, mostly protected by any dangerous interference.^16,25,26^ Of course, a certain degree of strength will be exerted by muscles (the tongue and cheek) on the implant, and the patient will be able to chew on it; however, the load and stress transmitted to the implant during the first healing period will be reduced and more controlled.^10,12,25,26^

To date, immediate loading has increasingly been popular among dentists worldwide and implant manufacturers are committed to providing surgeons with solutions that can meet their surgical and prosthetic needs.^10-13,15,25,26^ Specifically, new implant designs and macrotopographies are being studied to optimize the primary stabilization of the implant at positioning,^11,13,17,20,23^ while at the same time, new superficial micro- and nanotopographies are proposed to accelerate and increase, even in the first weeks of healing, the deposition of new bone on the implant surface.^21-24^

However, although there are many clinical studies on the immediate functional loading of rehabilitations such as full-arch mandibular prostheses,^27^ Toronto bridges^28^ and overdentures^15^ (prosthetic solutions wherein the implants are split between them, allowing better distribution of prosthetic load), few studies are currently available on the immediate functional loading of single, unsplinted implants.^13,29-34^

In particular, there are very few clinical studies available on the immediate functional loading of single implants in the posterior areas of the jaws, where the prosthetic load is greater, with all the risks involved.^10,13,31,33^ In fact, most of the papers in the literature present short-term results obtained through immediate loading of single implants placed in the anterior areas of the jaws, where the prosthetic load is lower.^6,9,32,34^

Given the lack of data and the weakness of the evidence in the literature,^29,30,32-34^ in 2012 we started a prospective multicentre study to identify the survival and success rates of single implants, placed in healed or post-extraction sites of both jaws and subjected to immediate functional loading. This clinical research led to the publication of two articles, which reported the results obtained with this protocol at 1 year^31^ and 2 years,^13^ respectively.

The aim of the present paper, therefore, is to present the 4-year results obtained in this study protocol, with immediately loaded single implants mainly located in the posterior areas of the jaws.

## Methods

### Study design, inclusion and exclusion criteria

This work was conceived and designed as a prospective and multicenter clinical study on patients treated with single crowns supported by implants and subjected to immediate functional loading.^13,31^ All in all, six clinical centers were involved in the present study, including two university centers and four private dental clinics. All the centers followed the same surgical and prosthetic protocol, which was defined in details *a priori*, that is, before the start of recruiting patients. Recruitment of patients and performance of the surgeries took place between February 2012 and February 2013, according to specific inclusion and exclusion criteria.

Inclusion criteria for the present study were as follows:

• patients ≥18 years of age

• patients with good general and oral health

• patients with single tooth gaps

• patients with one or more irreparably compromised single tooth/teeth to be extracted and replaced with a dental implant

• sufficient alveolar bone to insert an implant with a minimum length of 10.0 mm and a minimum diameter of 3.5 mm

• patients able to understand and sign an informed consent form for implant treatment

The exclusion criteria consisted of the following:

• patients with poor general health conditions (diabetic patients with poor glycemic control, severely immunocompromised patients, patients undergoing chemotherapy or radiotherapy for head and neck malignancies, patients treated with oral or parenteral aminobisphosphonates, patients with psychological or psychiatric disorders, patients with alcohol or drug addiction)

• patients with poor oral health conditions (patients with chronic periodontal disease with advanced bone loss, with active dental infections with pain, pus, fistula, and patients with oral pathologies)

• patients who needed to undergo major bone regeneration procedures before being able to receive dental implants (Minor regenerative procedures with granules of biomaterials such as coverage of exposed implant threads or protection/filling of vestibular and interproximal gaps were not criteria of exclusion for the present study.)

• patients who exhibited damage of the buccal bone wall of the extraction socket, following the extraction of a compromised tooth

• patients who did not have teeth in the opposing arch (and therefore did not have occlusal contacts)

• patients with parafunctions such as bruxism or clenching (The diagnosis of parafunction was carried out after anamnesis, objective examination and electromyography.)

Smoking was not an exclusion criterion for the present study, but all the enrolled patients were informed that cigarette smoking is a risk factor in implant therapy.^35^ All the patients were informed about the nature of the present study and signed a specific informed consent form for implant treatment. The study was conducted in full compliance with the Helsinki Declaration of 1975 (2000 revision) and received Ethics Committee approval at the University of Varese (approval #0034086).

### Surgical and prosthetic procedures

Each case was carefully planned through objective examination and radiographic evaluation with intraoral periapical and/or panoramic radiographs. Then, if the surgeon deemed it necessary, the patient was subjected to a three-dimensional (3D) evaluation with cone-beam computed tomography (CBCT). Such evaluations were necessary for the assessment of the residual bone structure in two and three dimensions, respectively. The DICOM (digital imaging and communication in medicine) files acquired with CBCT were also imported in a three-dimensional reconstruction software, to be able to evaluate in detail, in the three dimensions, the quantity and quality of residual bone. This was particularly important in patients with irreparably compromised single teeth, requiring dental extraction and immediate post-extraction implant placement. Evaluation of each patient was supplemented by generic impressions, stone casts and diagnostic wax-ups, useful for studying the ideal prosthetic solution.

In order to reduce the bacterial charge within the oral cavity, patients rinsed with 0.12% chlorhexidine twice or three times daily in the two days prior to the intervention, each rinse for 1 minute. The same procedure was repeated 15 minutes before the intervention. Surgery was performed under local anesthesia with articaine with 1:100.000 adrenaline.

In the case of patients with single tooth gap(s), a crestal incision was associated with two small vestibular release incisions. Then, the surgeon raised a full-thickness flap and started with osteotomy. This way, the surgeon performed an initial clinical evaluation of the bone quality of the recipient site. Under abundant saline irrigation, the surgeon then proceeded to prepare the implant site with drills of incrementally larger diameters. Once the implant site preparation was completed, the surgeon placed the implant of selected diameter and length in the surgical site. The fixture was positioned at the bone crest level. Immediately after the placement, the surgeon clinically checked the primary stability of the implant. However, in patients with one or more irreparably compromised teeth requiring extraction and replacement with an implant, the procedure was carried out without the elevation of a surgical flap. The irreparably compromised tooth was extracted with delicacy, being careful not to damage any of the four walls of the surgical alveolus. Indeed, the damage of only one of the four walls of the post-extraction socket was a criterion for exclusion of patients from the present study. The post-extraction socket was then curetted to remove any residual granulation tissue and, before implant placement, the surgeon checked the integrity of the four alveolar walls with a periodontal probe. At this point, the procedure continued with the preparation of the site via a pilot drill, engaged palatally, and apically in the socket for a minimum of 3‒4 mm, in order to optimize the primary stabilization of the implant. Then the preparation of the implant site was completed with drills of increasing diameter, as previously described.^13^ The whole procedure took place under abundant saline irrigation. The choice of the final diameter of the preparation was made according to the bone quality of the recipient site. At this point, the surgeon placed the implant, trying to avoid contact with the buccal bone wall. Following suggestions in the current literature,^36^ the implant was positioned in the palatal portion of the surgical socket, slightly below the crestal level. All the immediate implants were manually inserted slightly below the bone crest.

The implants used in this study were tapered (Anyridge®, Megagen Corporation, Gyeongbuk, South Korea), with strong self-cutting threads (KnifeThread®) to improve primary fixation^11,13,17,31^ and with a nanostructured calcium-incorporated surface (Xpeed®) to stimulate faster new bone apposition for secondary stabilization.^22,23^ The implants possessed an internal conical connection, and were available in different diameters (3.5, 4.0 and 4.5 mm) and lengths (10.0, 11.5, and 13.0 mm).^11,13,31^

Immediately after placement, the surgeon filled the remaining space between the fixture and the buccal bone wall and any remaining spaces between the implant and the mesial and distal bone walls of the socket with hydroxyapatite and beta tricalcium phosphate granules. This regenerative material could also be prophylactically applied over any exposed threads in the case of implants placed in healed sites as well as in post-extraction sites.

After the surgical phase and placement of the implant, a pre-fabricated titanium abutment was prepared and screwed onto the implant. All the provisional restorations were delivered within 48 hours of the intervention. They were delivered immediately if they were obtained from pre-formed shells, or within the next 48 hours if they were manufactured by the laboratory, after a polyvinilsilossane/polyether impression. In the case of pre-formed shells, the temporary crowns were adapted on the same abutment and relined with light-curing flowable resin. Relining could, however, be avoided in the case of provisional crowns fabricated by the laboratory. In any case, all the crowns were meticulously finished and polished, in order to obtain the best possible emergency profile for that restoration. In the group of implants placed in the healed ridge, the flap was adapted to the emergency profile and sutured with single interrupted sutures. In the post-extractive implant group, the provisional restorations sealed and maintained the clot formation subgingivally. Provisional crowns, each of which had a hole in the occlusal surface closed with composite resin, were screw-retained. The occlusion was meticulously controlled with articulating papers, and well-distributed occlusal contacts were obtained. In all the cases articulating papers were firmly held by the occluding teeth. An intraoral periapical radiograph was obtained with the temporary crown in position and, after that, the patients could be discharged.

The patients were discharged with analgesics and antibiotic prescription (100 mg nimesulide, every 12 hours, for 2 days and amoxicillin + clavulanic acid, 2 g per day, for 6 days) and were required to avoid chewing hard foods in the intervention area for a period of 2 weeks.

The first control was scheduled at 10 days after surgery for removal of the sutures (in the group of implants placed in the healed ridges) and the second 3-month control at the time of transition from the provisional crown to the final crown. The definitive restorations were fabricated in the laboratory, following a precise impression, and they were metal‒ceramic or ceramic crowns. They could be screwed or cemented. Again, an intraoral periapical radiograph of the implant was performed to control the adaptation of the final restoration. The patients were enrolled in a control program with a minimum of 2 professional oral hygiene sessions per year.

### Outcome variables

The outcome variables of the present study were basically three: implant survival, complications and peri-implant marginal bone loss (PIMBL). They were analyzed at different times: after the delivery of the provisional (T0) restoration, at the delivery of definitive restoration (T1; i.e. approximately 3 months after surgery/functional loading), and at 1 (T2), 2 (T3), 3 (T4) and 4 (T5) years from implant placement, respectively.

*•* **Implant survival:** The stability of each fixture was checked by applying a reverse torque of 20 Ncm. The stability was checked three times: at delivery of provisional and final crowns, and after 4 years of loading.

*•* **Complications:** Biologic complications were pain and/or swelling after surgery, peri-implant mucositis, and peri-implantitis. Prosthetic complications were abutment screw loosening and/or fracture and fracture of the ceramic veneer.

*•* **PIMBL:** The PIMBL was calculated as previously described.^13,31^ Basically, intraoral periapical radiographs were taken at different times (T0, T1, T2, T3, T4 and T5) for each implant, using a Rinn alignment system with a rigid film‒object X-ray source coupled to a beam-aiming device to achieve reproducible exposure geometry. Mesial and distal marginal bone levels of all the fixtures were measured at different time intervals (T0, T1, T2, T3, T4 and T5) and all the measurements were performed with the aid of an ocular grid, under ×4.5 magnification.

### Statistical evaluation

Data was collected by an independent operator who was not directly involved in the placement of fixtures and analyzed by the same operator. Statistical analysis included a descriptive part, evaluating the patient demographics (sex, age and smoking habit) and the features of the inserted fixtures systems (site, position, length, diameter and bone quality at the recipient site). Absolute and relative frequency distributions were calculated for qualitative variables, while means, standard deviations, medians and 95% confidence intervals (CI) were estimated for quantitative variables such as PIMBL. Implant survival and the incidence of biologic and prosthetic complications were calculated at both the patient and the implant levels, whereas PIMBL was calculated at the patient level only.

## Results

Forty-six patients (23 men and 23 women, between 18 and 73 years of age, with a mean age of 44.5 years) were selected for the present study. Data on patients enrolled in the present study (i.e. distribution by gender, age class and smoking habit), with relative drop-outs, failures and survival rates at 4 years are summarized in Table 1, while data related to the implants placed (i.e. distribution per site, position, length and diameter, together with the quality of the recipient sites) with related drop-outs, failures and survival rates at 4 years are summarized in Table 2. Overall, five patients had multiple indications for implant treatment, receiving more than one single implant. Fifty-seven implants were placed. The vast majority of implants (47/57, 82.5%) were placed in healed sites, while only 10 fixtures (10/57, 17.5%) were inserted into post-extraction sockets. In the latter case, the reasons for which the surgeon had to extract the compromised tooth and replace it with an implant were root fracture (7 fixtures, 70%), endodontic failure (2 fixtures, 20%), and extensive tooth decay (1 fixture, 10%). Among patients treated with immediate implant placement, no one was excluded because of damage of one or more of the socket walls during extraction. Surgeons did not have to perform bone regeneration procedures in most cases, as in only 15 implants (26.3%) was it necessary to cover exposed threads and/or to graft the interproximal/buccal areas with a biomaterial owing to hard tissue deficiency. The final restorations were screwed or cemented metal‒ceramic (52 cases, 91.2%) or zirconia‒ceramic (5 cases, 8.8%) crowns.

**Table 1:** Distribution of the patients by gender, age classes, smoking habit, with the related survival rates (patient-based)

	**No. of patients**	**Drop-outs**	**Failures**	**Survival rate (4 years)**
**Gender**
**Male**	23 (50%)	3	-	100%
**Female**	23 (50%)	2	1	95.3%
**Age**
**16‒25**	7 (15.2%)	-	-	100%
**26‒35**	6 (13.0%)	1	-	100%
**36‒45**	7 (15.2%)	1	-	100%
**46‒55**	13 (28.2%)	1	1	91.7%
**56‒65**	9 (19.5%)	1	-	100%
**> 65**	4 (8.7%)	1	-	100%
**Smoking habit**
**Smokers**	17 (37.0%)	2	1	93.4%
**Non-smokers**	29 (63.0%)	3	-	100%
**Overall**
**All patients**	46 (100%)	5	1	97.6%

**Table 2:** Distribution of the implants by site, position, length, diameter, bone type, with related survival rate (implant-based)

	**No. of implants**	**Drop-outs**	**Failures**	**Survival rate (2 years)**
**Site**
**Maxilla**	38 (66.7%)	3	1	97.2%
**Mandible**	19 (33.3%)	2	-	100%
**Position**
**Incisors**	9 (15.8%)	1	-	100%
**Cuspids**	3 (5.2%)	-	-	100%
**Premolars**	31 (54.4%)	2	1	96.5%
**Molars**	14 (24.6%)	2	-	100%
**Length**
**10 mm**	30 (52.7%)	2	1	96.5%
**11.5 mm**	21 (36.8%)	2	-	100%
**13 mm**	6 (10.5%)	1	-	100%
**Diameter**
**3.5 mm**	25 (43.9%)	1	1	95.8%
**4.0 mm**	21 (36.8%)	2	-	100%
**4.5 mm**	11 (19.3%)	2	-	100%
**Bone quality**
**Type II bone**	18 (31.6%)	1	-	100%
**Type III bone**	31 (54.4%)	2	1	96.5%
**Type IV bone**	8 (14.0%)	2	-	100%
**Overall**
**All implants**	57 (100%)	5	1	98.1%

**Figure 1:**
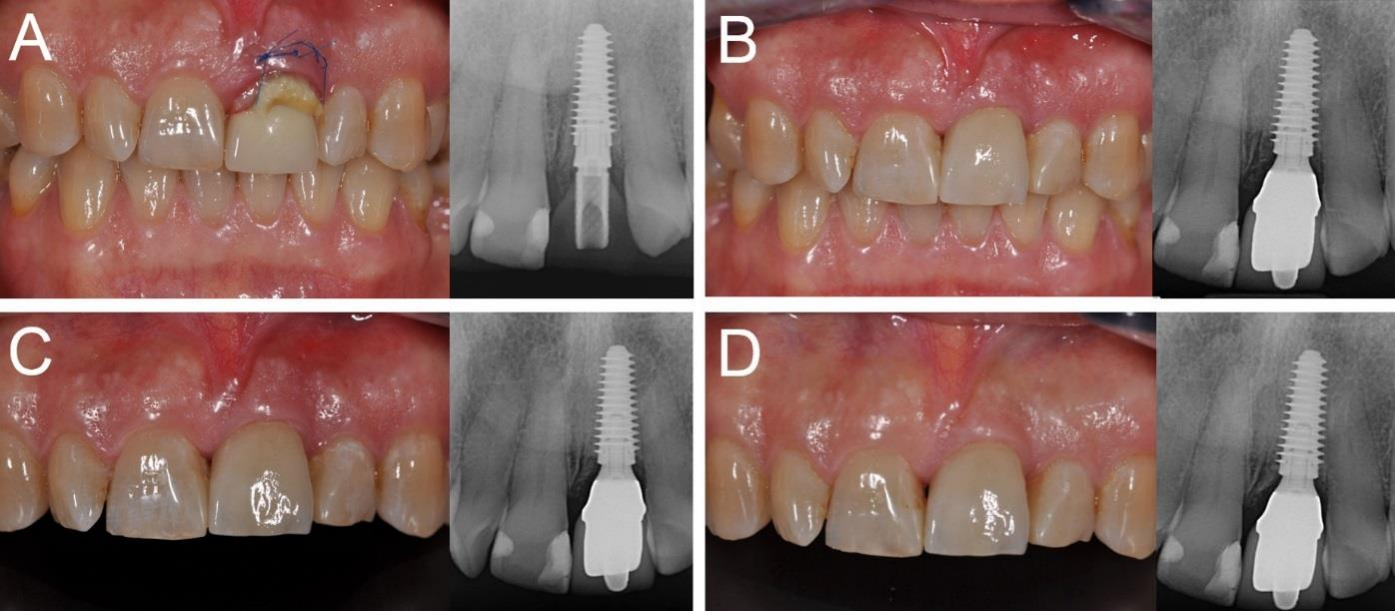
Immediate loading of a central maxillary incisor (#21) placed in a fresh extraction socket: (A) The implant is placed immediately after tooth extraction and loaded immediately after surgery; (B) three months later, the final metal‒ceramic crown is delivered; (C) the 1-year clinical and radiographic control; (D) the 4-year clinical and radiographic control.

**Figure 2:**
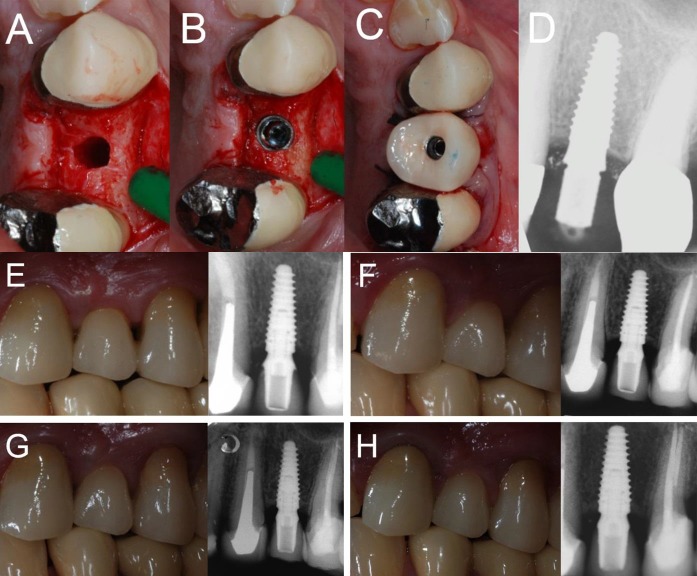
Immediate loading of a first maxillary premolar (#24) placed in a healed ridge: (A) The implant site is prepared; (B) the implant is placed at the bone crest level; (C) the provisional restoration is delivered immediately after implant placement; (D) peri-apical radiograph taken immediately after implant placement; (E) delivery of the final ceramic crown; (F) 1-year clinical and radiographic control; (G) 2-year clinical and radiographic control; (H) 4-year clinical and radiographic control.

**Figure 3:**
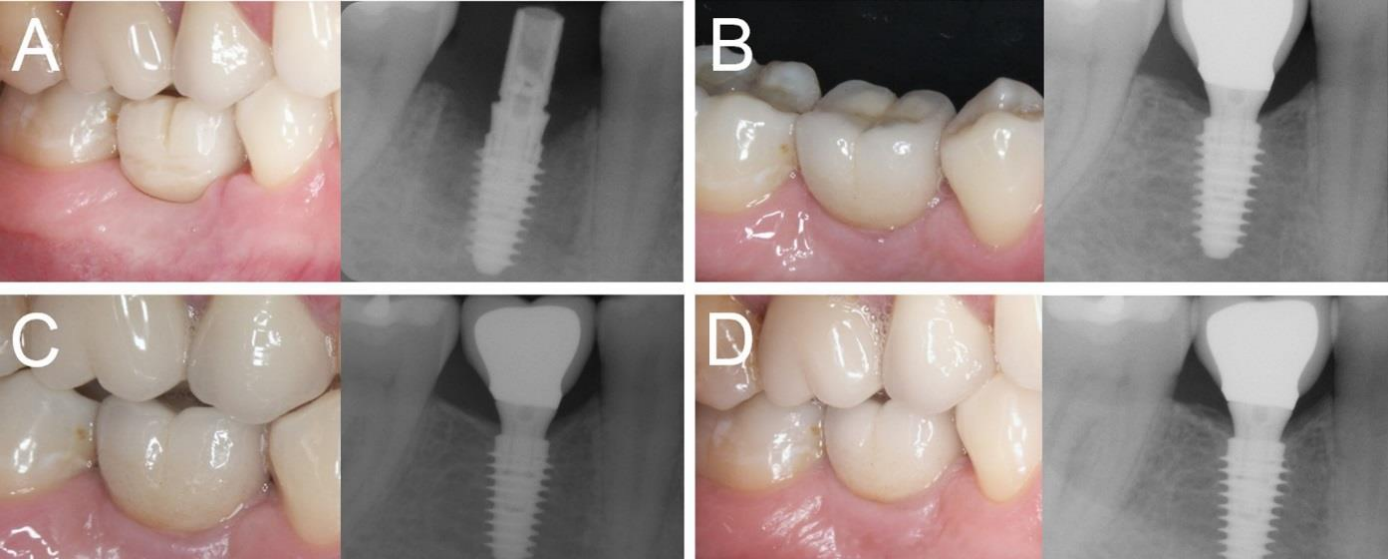
Immediate loading of a first mandibular molar (#46) placed in a healed ridge: (A) The implant is placed and loaded within 48 hours of the surgery; (B) three months later, the final metal-ceramic crown is delivered; (C) the 1-year clinical and radiographic control; (D) the 4-year clinical and radiographic control.

**Figure 4:**
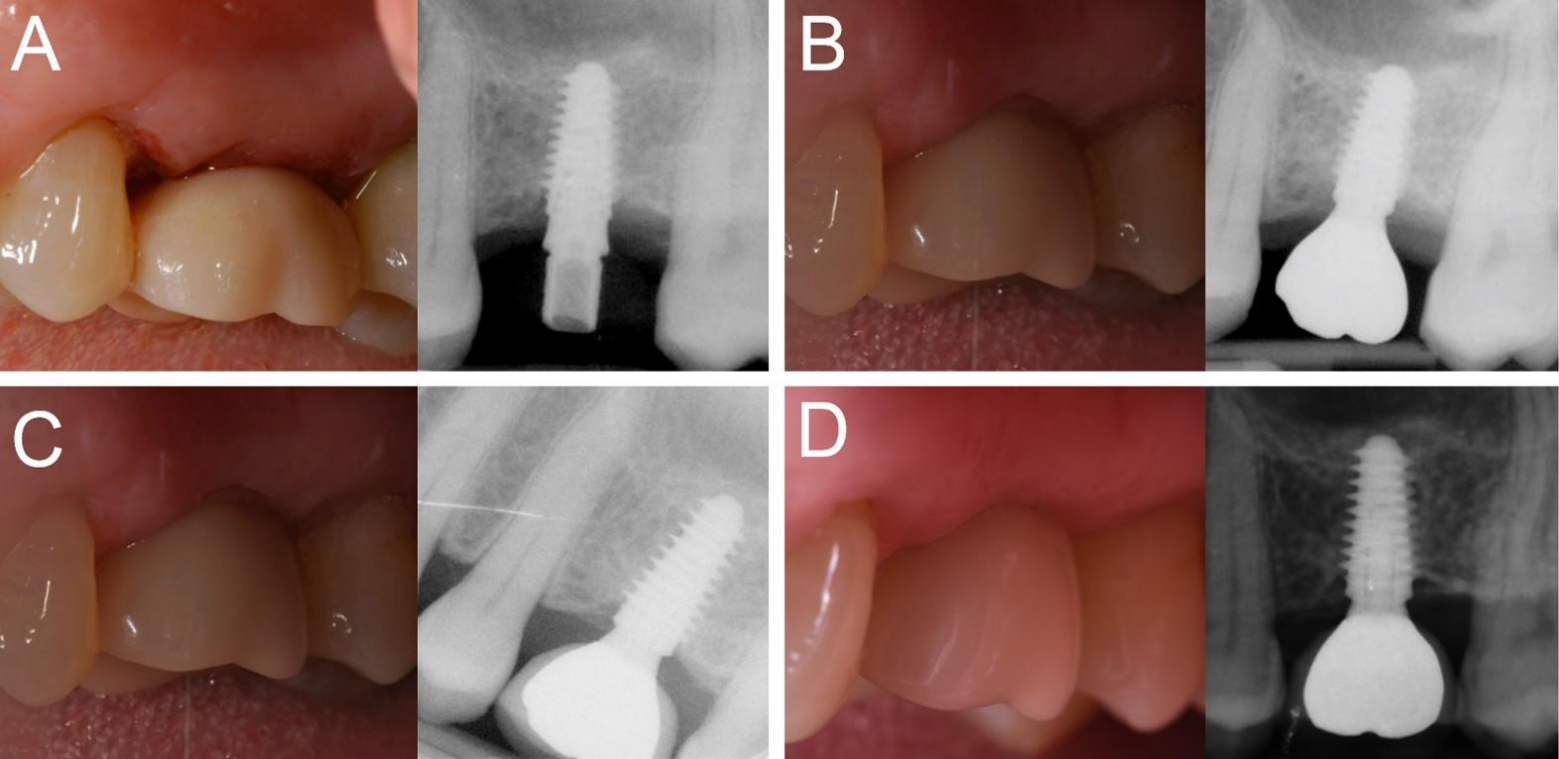
Immediate loading of a first maxillary molar (#26) placed in a healed ridge: (A) The implant is placed and loaded within 48 hours of the surgery; (B) three months later, the final metal‒ceramic crown is delivered; (C) the 1-year clinical and radiographic control; (D) the 4-year clinical and radiographic control.

Overall, five patients could not attend the 4-year control visit and were therefore classified as drop-outs, despite the fact that the implants were still in operation. However, 4 years after insertion, only one implant was lost, in the posterior maxilla (second premolar, healed site) of a smoking woman, 48 years old at the time of surgery. The failed implant was 3.5 mm in diameter and 10.0 mm in length and was installed in bone type III. This implant failed during the first healing period, exactly two months after the insertion and immediate functional loading, as it lost stability, in the absence of infection. All the other implants were stable; therefore, the overall 4-year implant survival rate was 97.6% (patient-based, with 40/41 fixture in the survival category) and 98.1% (implant-based, with 51/52 implants in the survival category), respectively (Figures 1‒4).

With regard to complications, two patients experienced biologic complications. One suffered from mild pain and swelling immediately after surgery and another suffered from peri-implantitis 3 years after implant placement. With regard to the first patient, the postoperative pain/swelling was simply managed, by prescribing analgesic medication, and no further discomfort was reported in that case. With regard to the patient who experienced peri-implantitis, it must be pointed out that he was a smoker with poor compliance for oral hygiene; he received a professional oral hygiene treatment with 4 sessions per year, and implant failure was avoided. The incidence of biologic complications was therefore 4.8% (patient-based, with 2/41 patients experiencing complications) and 3.8% (implant-based, with 2/52 implants showing complications), respectively.

The incidence of prosthetic complications was slightly higher (4/41 patients, 9.7%; 4/52 implants, 7.6%) than that of biological ones. Three patients (3/41 patients, 7.3%; 3/52 implants, 5.7%), in fact, presented with loosening of the prosthetic abutment (mechanical complications, minor in nature since they required the clinician to simply tighten the implant-abutment connecting screw), and one patient (1/41 patients, 2.4%; 1/52 implants, 1.9%) had a fracture of the ceramic veneer in a metal‒ceramic crown. This technical complication was considered a major one, since it forced the clinician and the dental technician to remake the work.

Finally, the PIMBL was as reported in Table 3. Overall, the PIMBL after 4 years of loading amounted to 0.38 mm (±0.21 mm; median: 0.4 mm; 95% CI: 0.32‒0.44 mm). In healed sites, the PIMBL was 0.4 mm (±0.21 mm; median: 0.4 mm; 95% CI: 0.33‒0.47 mm). In post-extraction sockets, the PIMBL was 0.33 mm (±0.20 mm; median: 0.4 mm; 95% CI: 0.21‒0.45 mm).

**Table 3:** PIMBL between groups at different time periods (patient-level), in mm

	**Baseline‒3 months**	**Baseline‒1 year**	**Baseline‒2 years**	**Baseline‒4 years**
	N, mean (SD), median, CI (95%)	N, mean (SD), median, CI (95%)	N, mean (SD), median, CI (95%)	N, mean (SD), median, CI (95%)
**Healed sites**	35; 0.23 (±0.18); 0.2; 0.18‒0.28	33; 0.36 (±0.21); 0,4; 0.29‒0.43	31; 0.4 (±0.22); 0.4; 0.33‒0.47	30; 0.4 (±0.21); 0.4; 0.33‒0.47
**Extraction sockets**	10; 0.20 (±0.18); 0.25; 0.09‒0.31	10; 0.22 (±0.20); 0.25; 0.10‒0.34	10; 0.3 (±0.22); 0.35; 0.17‒0.43	10; 0.33 (±0.20); 0.4; 0.21‒0.45
**All sites**	45; 0.22 (±0.17); 0.2; 0.18‒0.26	43; 0.33 (±0.22); 0.4; 0.27‒0.39	41; 0.37 (±0.22); 0.4; 0.31‒0.43	40; 0.38 (±0.21); 0.4; 0.32‒0.44

## Discussion

To date, there are very few studies on the immediate functional loading of single implants.^13,26,29-34^ Most of the studies in the literature show the results obtained with single implants after immediate non-functional loading (or immediate restoration) in the anterior areas with high aesthetic impact.^6,9,32,34^ This procedure, however, is certainly characterized by lower risks, as the crown is drained by the occlusion; the forces exerted on the implant are therefore lower than those present with immediate functional loading.^32,34^

Moreover, it should be noted that amongst the studies on the immediate functional loading of single implants, most of the results have been obtained with fixtures located in the anterior areas, subjected to lower masticatory forces;^32,34,37^ very few studies are available in which the implants have been placed mostly in the posterior areas (premolar and molars)^29,30,33^ and in which implants are inserted in fresh post-extraction sockets.^29,30^

A recent, randomized and controlled clinical trial on single implants compared the results obtained with two different surgical procedures and loading protocols: immediate functional loading after placement of fixtures with a flapless procedure versus delayed loading after placement of implants raising a surgical flap.^29^ At the end of the study, flapless surgical technique and immediate functional loading did not seem to compromise the survival and success of the implants.^29^

These findings were further confirmed in a subsequent clinical study with 4-year follow-up in which single implants inserted in the posterior areas of the jaws without raising a surgical flap and subjected to immediate functional loading exhibited high survival and success rates.^30^

In another prospective study, 40 single implants placed in the posterior mandible of 33 patients and subjected to immediate functional loading had a cumulative survival of 95% at 5 years, with only 2 registered implant failures.^33^

Excellent results are then reported in the literature for single implants placed in the anterior maxilla, and subjected to immediate loading.^6,9,32,34,37^

In a study of 70 patients treated with single implants placed in healed sites (45 fixtures) and in fresh post-extraction sockets (25 fixtures), 1 year after placement all the implants were in function, with no failures.^32^

Likewise, excellent results were reported in a clinical study on single implants undergoing immediate functional loading in aesthetic areas, with a 96.1% survival rate at 3 years and limited bone resorption.^34^

Thus, in agreement with a previous review of literature with meta-analysis, which reported on single implants placed in the anterior areas of the jaws, different loading protocols do not determine differences in implant survival and success rates.^37^

The results of the present clinical study, which represent the prospective evolution of our two previously published studies^13,31^ and which show the 4-year results of single implants mainly inserted into the posterior areas of the jaws and subjected to immediate functional loading, appear to be in agreement with what is reported in the literature.^13,29-34^ In fact, in over 57 implants positioned in 46 patients and after 4 years of follow-up, only one fixture was lost in the posterior maxilla (second premolar, healed site) of a smoking patient; this failure occurred in the first healing period and, subsequently, no further failures occurred during a 4-year period. After adjusting for the drop-outs that occurred, the overall 4-year implant survival rate was 97.6% (patient-based, with 40/41 fixtures in the survival category) and 98.1% (implant-based, with 51/52 fixtures in the survival category), respectively. These results seem to suggest that there are no differences in the survival rates of immediately loaded single implants placed in healed sites and post-extraction sites; however, only 10 implants were placed in post-extraction sites. In addition, in the present study, a low incidence of biologic complications was reported. In fact, only 2 patients experienced biologic complications, because one suffered from mild pain and swelling immediately after surgery and another suffered from peri-implantitis 3 years after implant placement. The moderate swelling and postoperative pain of the first patient was easily controlled by prescribing analgesics and disappeared after 4 days of implant insertion; similarly, the most feared biological complication of peri-implantitis occurring in the second patient at 3 years of placement was successfully controlled and limited through a series of professional oral hygiene sessions. Four years after placement, therefore, the incidence of biologic complications was 4.8% (patient-based, with 2/41 patients experiencing complications) and 3.8% (implant-based, with 2/52 implants showing complications), respectively. The incidence of prosthetic complications in the present 4-year work was relatively higher; in fact three patients (3/41 patients, 7.3%; 3/52 implants, 5.7%) experienced loosening of the prosthetic abutment and one patient (1/41 patients: 2.4%; 1/52 implants, 1.9%) had a fracture of the ceramic veneer. In view of these minor mechanical and major technical complications, the overall incidence of prosthetic complications in our study was 9.7% (patient-based, with 4/41 patients experiencing complications) and 7.6% (implant-based, with 4/52 implants experiencing complications), respectively.

It should be noted, finally, how the differences between peri-implant bone resorption reported in the current 4-year clinical trial and in our previously published 2-year^13^ and 1-year^31^ studies are really minimal, indicating considerable stability of peri-implant hard tissues over time. In fact, in the present clinical study, we found an overall 4-year PIMBL of 0.38 mm (±0.21 mm; median: 0.4 mm; 95% CI: 0.32‒0.44 mm). In healed sites, the PIMBL was 0.4 mm (±0.21 mm; median: 0.4 mm; 95% CI: 0.33‒0.47 mm). In post-extraction sockets, the PIMBL was 0.33 mm (±0.20 mm; median: 0.4 mm; 95% CI: 0.21‒0.45 mm). Compared to what was reported after 2 years of functional loading,^13^ the difference was minimal.

In the immediate functional loading procedures, the determining factors for success are several: the bone quality of the recipient site, the primary stabilization of the implant through adequate implant bed preparation, the presence of a controlled load, and the macro-, micro- and nano-topographical characteristics of the fixture used.^11,13,17,20-23,31, 38-40^ The quality of the recipient site is key, and the immediate functional loading of a single implant in the posterior maxilla is considered to be at greater risk of failure than the same procedure in the anterior areas or in the posterior mandible. Primary stability is the biometric stability achieved immediately after implant insertion, by the mechanical locking of the implant to the bone, and it is essential. It certainly depends on the quality of the recipient bone, but also on the surgical protocol used, the ability and experience of the surgeon, and not least on the macro-topographical characteristics and design of the implant used.^17-20^ A good trick to stabilize the implant is to underprepare the receiving site, and this is especially true for fixtures located in post-extractive sites, where primary stabilization is generally obtained in the 3‒4 mm of bone apical to the fresh extraction socket.^6,9,36^ Obviously, the use of a conical implant with an aggressive thread design can help achieve good primary stabilization, even in difficult sites, as evidenced in the literature.^11,13,17,23,31,39,40^

In the present study, most of the implants were placed in the posterior areas of the jaw; hence the surgeons adhered to a strict surgical protocol in which the fixtures were placed in underprepared sites (especially in the case of post-extraction sockets). The surgeons had remarkable and extensive clinical experience in functional immediate loading. Finally, the implants used in this study were tapered with extremely aggressive threads, endowed with a macro-topography capable of maximizing stabilization upon insertion.^11,13,17,23,31,39,40^

It is important to note that most of the implants placed in the present study (26 fixtures; 45.7%) were inserted in the posterior maxilla, which is characterized by poor bone quality. In this area, micromovements beyond a critical threshold can lead to fibrous encapsulation of the implant and subsequent failure;^17-20,40^ however, only one failure was reported here.

Finally, in immediate loading procedures, it is important to use implants with appropriate micro- and nano-topographies, designed to accelerate and maximize bone healing processes and thus the secondary (biological) stabilization of the implant.^21-23^ In the present study, we have used implants characterized by peculiar micro- and nano-topographical features, with a novel nanostructured calcium-incorporated surface;^22,23^ this surface is highly osteoconductive and can promote bone healing, as demonstrated by different human histologic and histomorphometric studies.^22,23^ This micro- and nano-topography, combined with a tapered design with aggressive implant threads for critical bone conditions and high insertion torques^11,17,20,31^ may have minimized the risk of failure associated with immediate functional loading.

## Conclusions

In this multicenter prospective 4-year clinical study on the immediate functional loading of single implants, we found high survival rates (97.6% survival rate, patient-based; 98.1% survival, implant-based) and limited incidence of biological (4.8% patient-based; 3.8% implant-based) and prosthetic (9.7% patient-based; 7.6% implant-based) complications. In addition, the overall PIMBL was limited, with an average bone loss of 0.38±0.21 mm (healed sites: 0.4±0.21 mm; post-extraction sockets: 0.33±0.20 mm) after 4 years. The present study had some limitations, including the low number of treated patients and the low number of inserted implants. Further studies will therefore be necessary to confirm the results obtained.

**Acknowledgment**s

None

## Competing interest

The authors have no conflict of interests related to the publication of the present work.
